# Simultaneous targeting of linked loci in mouse embryos using base editing

**DOI:** 10.1038/s41598-018-33533-5

**Published:** 2019-02-07

**Authors:** Hye Kyung Lee, Michaela Willi, Harold E. Smith, Shannon M. Miller, David R. Liu, Chengyu Liu, Lothar Hennighausen

**Affiliations:** 1 Laboratory of Genetics and Physiology, National Institute of Diabetes and Digestive and Kidney Diseases, US National Institutes of Health, Bethesda, Maryland 20892 USA; 2 Genomics Core, National Institute of Diabetes and Digestive and Kidney Diseases, US National Institutes of Health, Bethesda, Maryland 20892 USA; 3 Merkin Institute of Transformative Technologies in Healthcare, Broad Institute of Harvard and MIT, Cambridge, Massachusetts 02142 USA; 4 Howard Hughes Medical Institute, Harvard University, Cambridge, MA 02138 USA; 5 Department of Chemistry and Chemical Biology, Harvard University, Cambridge, MA 02138 USA; 6 Transgenic Core, National Heart, Lung, and Blood Institute, US National Institutes of Health, Bethesda, Maryland 20892 USA

## Abstract

A particular challenge in genome engineering has been the simultaneous introduction of mutations into linked (located on the same chromosome) loci. Although CRISPR/Cas9 has been widely used to mutate individual sites, its application in simultaneously targeting of linked loci is limited as multiple nearby double-stranded DNA breaks created by Cas9 routinely result in the deletion of sequences between the cleavage sites. Base editing is a newer form of genome editing that directly converts C∙G-to-T∙A, or A∙T-to-G∙C, base pairs without introducing double-stranded breaks, thus opening the possibility to generate linked mutations without disrupting the entire locus. Through the co-injection of two base editors and two sgRNAs into mouse zygotes, we introduced C∙G-to-T∙A transitions into two cytokine-sensing transcription factor binding sites separated by 9 kb. We determined that one enhancer activates the two flanking genes in mammary tissue during pregnancy and lactation. The ability to introduce linked mutations simultaneously in one step into the mammalian germline has implications for a wide range of applications, including the functional analysis of linked *cis*-elements creating disease models and correcting pathogenic mutations.

## Introduction

Clustered regularly interspaced short palindromic repeat (CRISPR)-Cas9 genome editing^1,2^ has been widely used to disrupt individual and multiple targets^3–6^. However, co-targeting two or more sites on the same chromosome usually results in the excision of the DNA between targets^3,7–10^, especially for sites in close proximity (up to 1 Mb deletions have been reported)^8–10^. To tackle this particular technical challenge, we turned to base editing (BE)^11–13^ with its advantage of changing specific nucleotides in the genome without inducing double-strand breaks, which should make it less likely to cause undesired mutations, such as deletions or insertions. The deaminases currently in use facilitate the conversion of cytosine to uracil or adenosine to inosine and subsequent DNA repair results in a C∙G-to-T∙A or A∙T-to-G∙C substitution^11–13^. Thus, this approach should enable the simultaneous mutation of linked sites without causing deletions.

The introduction of mutations into linked loci is essential for experimental approaches to understand complex loci with multiple haplotypes of single-nucleotide polymorphisms (SNPs) related to diseases^14,15^, roles of individual enhancers within super-enhancers^7,16^, and tumorigenesis^17,18^. This approach is also needed to examine the possibility of correcting co-occurring somatic mutations^14,15^. Therefore, it is paramount to establish a reliable tool that permits the efficient and faithful introduction of two or more linked mutations in one step. In this study we have investigated the ability of cytosine base editors to simultaneously introduce point mutations in two sites separated by 9 kb. To ensure a sensitive and reliable readout, we chose to mutate enhancers that possibly activate genes in mammary tissue during pregnancy.

## Results

### Simultaneous targeting of linked loci by cytosine-deaminase-mediated base editing

First, we aimed to determine the extent of deletions introduced upon simultaneously targeting linked sites by CRISPR/Cas9. We co-microinjected two or more sgRNAs together with Cas9 mRNA into mouse zygotes. Although it is known that co-targeting two or more linked loci can result in the deletion of the entire sequence between the targets, we were surprised by the prevalence of big deletions spanning both sites compared to small deletions at each cutting site. Targeting two sites separated by 18 kb resulted in the complete deletion of the locus in 11 out of 14 founders (Supplementary Fig. 1a). In a second experiment, 15 out of 23 founder mice carried a complete deletion between target sites that were 9 kb apart (Supplementary Fig. 1a). Other studies have reported deletion sizes between 10 kb and 0.5 Mb at frequencies between 10% and 90% (Supplementary Fig. 1a and b). These results emphasize the extraordinary efficiency of CRISPR/Cas9 technology, but also suggest that Cas9 proteins may not function independent of each other. Deleting sequences between two sites requires that both Cas9/sgRNA complexes cut the same allele at exactly the same moment. Therefore, deletions should not occur if a cut introduced by Cas9 is repaired through non-homologous end joining (NHEJ) before the second cut is introduced. The surprisingly high efficiency of big deletions suggests that Cas9-created double strand breaks are not immediately repaired by NHEJ, or multiple Cas9 molecules may communicate with each other and cut DNA simultaneously.

**Figure 1:**
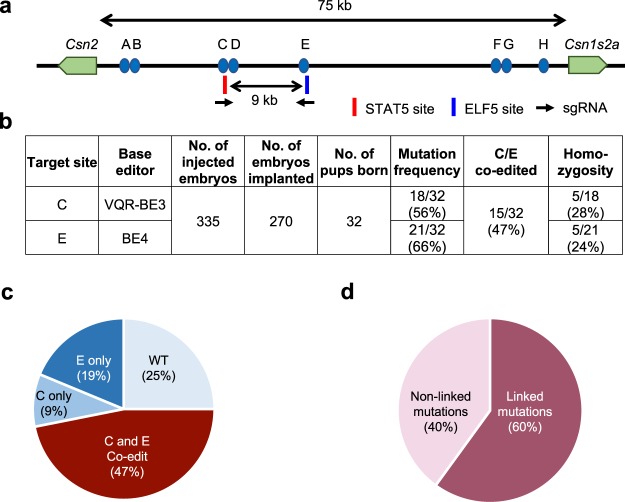
Simultaneously targeting of two linked genomic loci by cytosine-deaminase-mediated base editing. (**a**) Schematic diagram of target sites in the *casein* locus. The eight putative enhancers (A-H) and the two promoters in the *Csn2* and *Csn1s2a* locus were identified by ChIP-seq analysis for enhancer marks. Sites C and E are 9 kb apart and were targeted simultaneously with two sgRNAs and VQR-BE3 and BE4. (**b**) Summary of data obtained from mouse zygotes co-injected with VQR-BE3 and BE4 mRNA, and two sgRNAs. Experiments were conducted with founder mice and established mouse lines. (**c**) C-to-T mutation frequency observed at the two sites. (**d**) Distribution of linked (on the same chromosome) and non-linked mutations.

**Figure 2:**
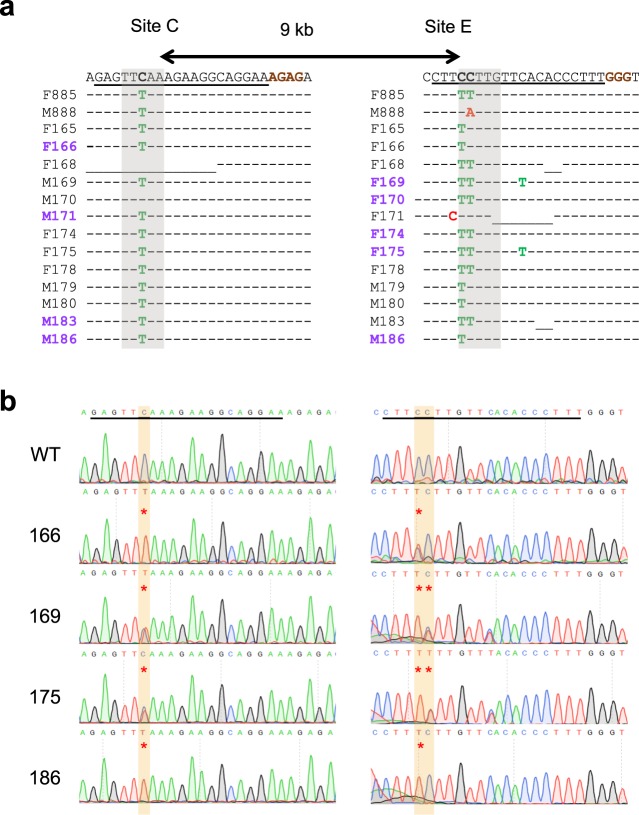
Alignment of sequences from founder mice carrying mutations in sites C and E. (**a**) sgRNA sequences are underlined and the PAM sites are shown in brown. The C-to-T on-target substitutions are shown in green. Mutant mice carrying homozygous mutations are marked in bold purple. Unintended nucleotide substitutions are shown in red. Deletions are shown as underlines. WT, wild-type. (**b**) Sanger sequencing chromatograms of DNA from WT and mutant mice carrying homozygous mutations (founder 166, 169, 175, and 186). The sgRNA sequences are underlined. C-to-T transitions are seen at target sites C and E and marked with a red asterisk.

**Figure 3:**
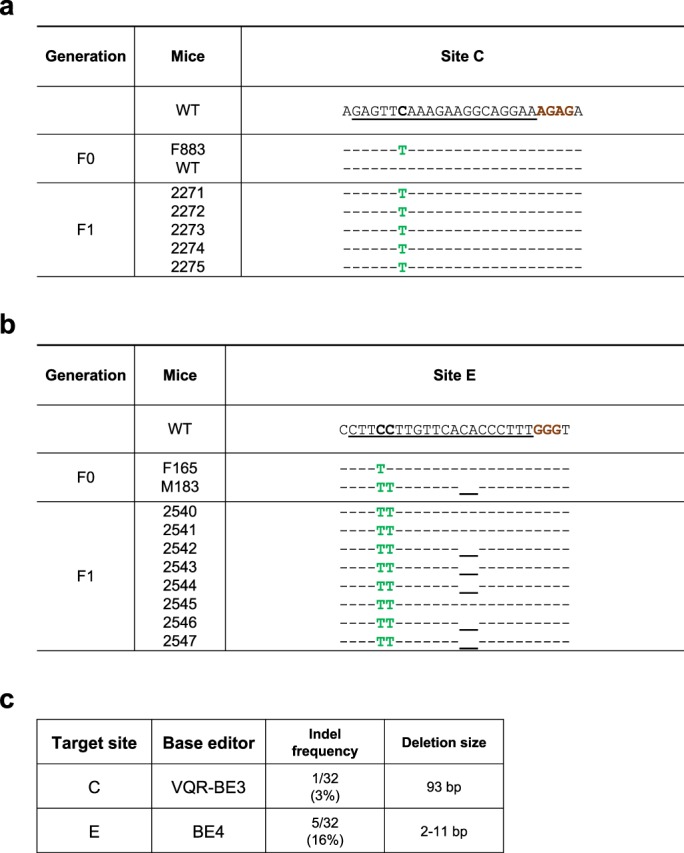
Inheritance of intended mutations. (**a**) Female founder F883 was mated with a WT male and mutations in site C were analyzed in its offspring. F883 was homozygous for the intended C-to-T conversion on site C and all offspring were heterozygous for this mutation. The C-to-T editing is shown in green. (**b**) F165 was mated with male founder M183, and intended mutations and deletions are co-segregated. (**c**) The indel frequency and deletion length were analyzed in founder mice.

Next, we investigated the ability of base editors to introduce mutations into linked loci without disrupting sequences between target sites. We focused on a 75 kb region in the mouse *casein* locus, which contains at least eight putative enhancers (A-H) (Fig. 1a). These enhancers were identified using ChIP-seq experiments and are characterized by the binding of transcription factors STAT5, GR, ELF5, MED1 and the presence of the active histone marker H3K27ac (Supplementary Fig. 2). The location of these putative enhancers infers a regulatory role in controlling expression of the associated *Csn2* and *Csn1s2a* genes during pregnancy and lactation. We used VQR-BE3, which recognizes a NGA PAM^19^, and BE4, which recognizes a NGG PAM^13^, to mutate transcription factor binding motifs in sites C and E, respectively. We co-injected VQR-BE3 and BE4 mRNAs and their corresponding guide RNAs, targeting the STAT5 motif (TTCNNNGAA) in site C and an ELF5 motif (GGAA/T) in site E (Fig. 1a and Supplementary Fig. 2), into mouse zygotes and transferred injected embryos into oviducts of pseudo-pregnant recipients. Out of the 32 founder mice, 9% carried target mutations exclusively in site C, 19% only in site E, and 47% carried target mutations in both sites (Figs 1b, 1c and 2a). Twenty-five percent of the founders did not carry any mutation (Fig. 1c). Homozygosity was prevalent with 28% of the founders at site C and 24% at site E (Figs 1b and 2b). In nine out of the 15 co-targeted founders, the mutations in sites C and E were linked, i.e. they co-located on the same homologous chromosome (Fig. 1d). Mutations were passed on through the germline (Figs 3a and b). Unlike conventional CRISPR/Cas9 genome editing, which results in the deletion of sequences between sites targeted by sgRNAs^3,7–10^ (Supplementary Fig. 1), we did not detect such deletions in any of the 32 founders and their offspring. However, we found indels around target sites and out of the 32 founder mice, one carried a 93 bp deletion at site C and five mice carried deletions between 2 to 11 bp at site E (Fig. 3c). Presumably, those deletions are the results of the nickase activity of BE on a single strand and cell’s endogenous DNA repair machinery^20^. Our results demonstrate that base editing, in contrast to CRISPR/Cas9 genome editing, can be used to simultaneously and efficiently introduce linked mutations in the mouse germline without disrupting the targeted locus.

### Enhancer mutations selectively impact neighboring genes

**Figure 4:**
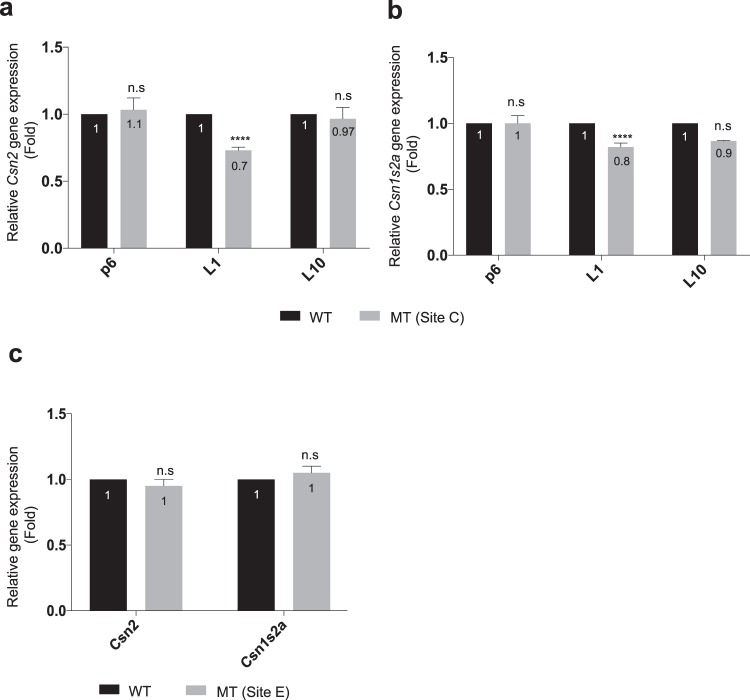
Biological consequence of enhancer mutations during pregnancy. (**a**–**c**) Expression of the *Csn2* and *Csn1s2a* genes in mammary tissue from WT and mutant mice carrying nucleotide substitution at site C or E, respectively, by qRT-PCR. mRNA levels were normalized to *Gapdh* levels. Results are shown as the means ± s.e.m. of independent biological replicates (WT and mutants, *n* = 3). *T-test* was used to evaluate the statistical significance of differences between WT and mutants. ****P < 0.00001. n.s., not significant.

Given the complexity of the enhancer landscape associated with the *Csn2* and *Csn1s2a* genes and the possibility of compensatory functions among the eight constituent enhancers (Fig. 1a, Supplementary Fig. 2), the biological consequences of the mutations were far from clear. *Csn2* and *Csn1s2a* mRNA levels increase 60- and 130-fold, respectively, during pregnancy^21^. To investigate whether the mutations in sites C and E curb the induction of these two genes, we measured their mRNA levels at day six of pregnancy (p6), and days one (L1) and ten (L10) of lactation. While *Csn2* expression at p6 was not affected by the mutation in site C, expression at L1 was reduced by 30% (Fig. 4a), revealing a contribution of this enhancer in gene activation during pregnancy. Expression of *Csn1s2a*, which is located 50 kb from site C, was affected to a lesser extent (Fig. 4b). *Csn2* expression at L10 was not curtailed by the mutation in site C suggesting a high degree of compensation between enhancers during lactation, similar to that observed in another mammary super-enhancer^7^. Mutations of an ELF5 site in enhancer E did not affect *Csn2* or *Csn1s2a* mRNA levels during pregnancy and lactation (Fig. 4c). Our findings lend biological meaning to an enhancer in the activation of two *casein* genes during pregnancy. The moderate activity of enhancer C within the complex *casein* super-enhancer is in line with other studies addressing genes under the control of multiple enhancers and super-enhancers, including *Wap* in mammary tissue^7^, *Il2ra* in T cells^16^, and *α-globin* in erythroid cells^22^.

### Off-target analysis by WGS

Finally, to assess off-target effects, we initially used computational prediction and identified potential off-target sites for each sgRNA, with up to 4-nucleotide mis-matches in the mouse genome (Supplementary Fig. 3a). A total of 434 potential off-target sites were identified for the sgRNA used with VQR-BE3 and 143 for the sgRNA used with BE4. To evaluate targeting at predicted off-target sites, we performed whole genome sequencing (WGS) of two founder mice carrying linked mutations in sites C and E and a cohort of mice^23^ from the same genetic background as controls. We did not detect any SNPs and indels at the predicted off-target sites (Supplementary Fig. 3b and Supplementary Table 1).

## Discussion

Our study addressed a major unresolved technical challenge and conclusively demonstrates that cytosine base-editing can be used to simultaneously and efficiently introduce linked mutations in mouse embryos without deleting sequences between the target sites. This experimental approach opens opportunities, both in basic and translational research, to address the biology of complex loci carrying several haplotypes. Our findings also provide biological significance to a constituent enhancer within a complex super-enhancer, which contributes to the activation of the *Csn2* gene during mammary differentiation^24^. Our data also suggest limited enhancer redundancy within this locus and a more complete understanding of its regulation will require the introduction of mutations into most, if not all of the eight constituent enhancers. Base-editing could be the preferred option and, depending on the editing window and the sequences of potential protospacer adjacent motif (PAM), the use of several different base editors recognizing different PAMs will be required.

## Methods

### Mice

All animals were housed and handled according to the guidelines of the Animal Care and Use Committee (ACUC) of the NIH (https://oacu.oir.nih.gov) and all animal experiments were approved by the ACUC of National Institute of Diabetes and Digestive and Kidney Diseases (NIDDK, MD) and performed under the NIDDK animal protocol K089-LGP-17. Base editing- and CRISPR/Cas9-targeted founder mice were generated using C57BL/6 N mice (Charles River Laboratories, MD) by the Transgenic Core of the National Heart, Lung, and Blood Institute (NHLBI, MD).

### Targeted loci

Using CRISPR/Cas9 genome editing, two sites in the *casein* locus that are 9 kb apart and two sites in *Wap*-*Ramp3* locus that are 18 kb apart, were co-targeted by Cas9 and two sgRNAs, respectively. In the 75 kb locus with 8 enhancers, transcription binding sites on sites C and E that were 9 kb apart were targeted simultaneously using VQR-BE3 and BE4 base editors, and two sgRNAs to introduce C-to-T transitions.

### mRNA preparation and microinjection into mouse zygotes

The sgRNAs were designed based on the nearest PAM of the target sequence. Each sgRNA was cloned into the pDR274 plasmid vector (Addgene #42250, MA), and *in vitro* transcribed using the MEGAshortscript T7 kit (ThermoFisher Scientific, MA). pCMV-VQR-BE3 and BE4 mRNAs was synthesized *in vitro* using the mMESSAGE mMACHINE T7 kit (ThermoFisher Scientific). Cas9 (100 ng/μl) and deaminase fused-Cas9 mRNA (50 ng/μl for each base editor) and sgRNAs (20 ng/μl for each sgRNA) were mixed and co-microinjected into the cytoplasm of fertilized eggs collected from superovulated C57BL/6 N female mice (Charles River Laboratories) and implanted into oviducts of pseudopregnant fosters (Swiss Webster, NY).

### Generation of V5-tagged ELF5 mouse

V5-tagged Elf5 mutant mouse by injecting the oligo donor with V5-tag and sgRNA into zygotes.

### Genotyping

Genomic DNA of all mice was isolated from the tip of the tail, amplified by PCR, and followed by Sanger sequencing. Large deletions were identified by serial PCR genotyping using primers that were designed to amplify 400~500 bp encompassing the target sequence or long-range PCR.

### RNA isolation and quantitative real-time PCR (qRT–PCR)

Total RNA was extracted from frozen mammary tissue of wild-type and mutant mice using a homogenizer and the PureLink RNA Mini kit according to the manufacturer’s instructions (Invitrogen, MA). Total RNA (1 μg) was reverse transcribed for 50 min at 50 °C using 50 μM oligo dT and 2 μl of SuperScript III (Invitrogen) in a 20 μl reaction. Quantitative real-time PCR (qRT-PCR) was performed using TaqMan probes (*Csn2*, Mm04207885_m1; *Csn1s2a*, Mm00839343_m1; mouse *Gapdh*, Mm99999915_g1, ThermoFisher scientific) on the CFX384 Real-Time PCR Detection System (Bio-Rad, CA) according to the manufacturer’s instructions. PCR conditions were 95 °C for 30 s, 95 °C for 15 s, and 60 °C for 30 s for 40 cycles. All reactions were done in triplicate and normalized to the housekeeping gene *Gapdh*. Relative differences in PCR results were calculated using the comparative cycle threshold (*C*_*T*_) method.

### Statistical analyses

Shapiro-Wilk normality test returned for all groups a p-value above 0.05. Thus, the hypothesis that the samples come from a population with normal distribution was not rejected. For comparison of samples, data were presented as standard deviation in each group and were evaluated with a *t-test* using PRISM GraphPad. Statistical significance was obtained by comparing the measures from wild-type or control group, and each mutant group. A value of *P < 0.05, **P < 0.001, ***P < 0.0001, ****P < 0.00001 was considered statistically significant.

### Chromatin immunoprecipitation sequencing (ChIP-seq) and data analysis

Mammary tissue was harvested at day one of lactation and stored at −80 °C. Frozen tissues were ground into powder in liquid nitrogen. Chromatin was fixed with formaldehyde (1% final concentration) for 15 min at room temperature, and then quenched with glycine (0.125 M final concentration). Samples were processed as previously described^25^. The following antibodies were used for ChIP-seq: V5 tag antibody (ThermoFisher Scientific, R960-25). Libraries for next-generation sequencing were prepared and sequenced with a HiSeq 2500 instrument (Illumina). Quality filtering and alignment of the raw reads was done using Trimmomatic^26^ (version 0.36) and Bowtie^27^ (version 1.1.2), with the parameter -m to keep only uniquely mapped reads, using the reference genome mm10. Picard tools (Broad Institute. Picard, http://broadinstitute.github.io/picard/. 2016) was used to remove duplicates and subsequently, Homer^28^ (version 4.8.2) software was applied to generate bedGraph files. Integrative Genomics Viewer^29^ (version 2.3.81) was used for visualization.

### Off-target analysis

Off-target sites were predicted using http://crispor.tefor.net/^30^. The resulting off-target sites were filtered using the same criteria as for SNPs and indels, to consider only those areas of the genome which do not coincide with black regions^31^ (ENCODE^31^; http://mitra.stanford.edu/kundaje/akundaje/release/blacklists/mm10-mouse) or repetitive elements^32^ (UCSC’s masked repeats plus simple repeats; http://hgdownload.soe.ucsc.edu/goldenPath/mm10/database). Mutations, which were present in the population and not only in base-edited mice, but identified at predicted off-target sites, were not considered as a consequence of base editing.

### GATK analysis

WGS (60×) was performed of two founder mice carrying a base substitution at site C and E using three guide RNAs and two base editors. In addition, we analyzed 30 mice as control, 24 wild-types mice (males and females) and six of their non-injected progeny. The analysis was done accordingly to the GATK best practices guidelines^33–35^ for germline mutations (version 3.8-0). Thus, BBmap^36^ (version 37.36) was applied for quality control, followed by BWA MEM^37^ (version 0.7.15) for the alignment step (reference genome mm10). The aligned BAM files were subsequently split up to a chromosome level (for runtime optimization) and reads aligned to different chromosomes were filtered using SAMtools^38^ (version 1.5). Additionally, Picard tools^39^ (version 2.9.2) was applied to mark duplicates. The subsequent GATK analysis workflow comprised: (i) base recalibration - GATK BaseRecalibrator, AnalyzeCovariates, and PrintReads - using the databases of known polymorphic sites, dbSNP142 and MGPv5 (provided by the high-performance computing team of the NIH (Biowulf)); (ii) variant calling - GATK HaplotypeCaller - with the genotyping mode “discovery”, the “ERC” parameter for creating gvcf and a minimum phred-scaled confidence threshold of 30. The final step included merging the VCF files of each chromosome (GenomeAnalysisTK, GATK).

### GATK SNP analysis

Joint genotyping was applied on each of the three groups independently (wild-type, non-injected controls and base-edited mice). On each of the groups hard filtering with the following parameters was applied: “QD < 2.0||FS > 60.0||MQ < 40.0||MQRankSum < −12.5||ReadPosRankSum < −8.0||SOR > 3”. The resulting SNPs were filtered by removing those overlapping with repetitive elements^32^ (UCSC’s masked repeats plus simple repeats; http://hgdownload.soe.ucsc.edu/goldenPath/mm10/database) and black regions (ENCODE^31^; http://mitra.stanford.edu/kundaje/akundaje/release/blacklists/mm10-mouse/). On an individual level those SNPs with a genotype of 0/1 or 1/1 were kept. Further filtering included the removal of SNPs with a read depth smaller than 10, an excessive read depth^40^ (d + 3√d, d = average read depth), an allele frequency less than 10% and a quality smaller than 130 using a variety of tools^41–43^. All SNPs within +/−5 bp of an indel border were removed as likely false-positives.

### Simple GATK indel analysis

Indels were extracted from the GATK joint genotyping file and hard filters were applied based on the GATK recommendations: “QD < 2.0||FS > 200.0||ReadPosRankSum < −20.0||SOR > 10.0”. Indels overlapping with repetitive elements^32^ (UCSC’s masked repeats plus simple repeats; http://hgdownload.soe.ucsc.edu/goldenPath/mm10/database) or black regions (ENCODE^31^; http://mitra.stanford.edu/kundaje/akundaje/release/blacklists/mm10-mouse/) were removed. Subsequently, each file was filtered keeping only indels with the genotypes of 0/1 and 1/1, removing those with a read depth smaller than 10 as well as sites with an excessive number of reads^40^ (d + 3√d, d = average read depth). The last step comprised the removal of all simple indels that overlap with complex indels identified using LUMPY. For all those steps a variety of tools^41–43^ was applied.

### Complex indel analysis using LUMPY

Indel analysis was done on the same samples as described above using Lumpy^44^ according to the guidelines. Thus, BWA MEM^37^, with the parameters “–excludeDups–addMateTags–maxSplitCount 2–minNonOverlap 20” was applied for mapping (reference genome mm10), followed by Lumpy^44^ using the discordant and split reads as input. Post-processing was carried out using SVTyper^45^ to identify genotypes. The filtering step comprised the selection of indels with a genotype of 0/1 and 1/1 and the removal of indels with a quality smaller than 100 and an excessive read coverage (d + 3√d^40^, where d is the average read depth). Indels overlapping with repetitive elements^32^ or black regions^31^ were excluded.

## Electronic supplementary material

Dataset 1

## Data Availability

ChIP-seq data of wild-type mammary tissue at L1 were obtained from GSE74826 and GSE115370 in the Gene Expression Omnibus (GEO). ChIP-seq for V5 tag have been deposited under GSE119657. The WGS data of the wild-type mice are available at SRA PRJNA470569. The WGS data of the base-edited mice are deposited at SRA PRJNA489707.
