# Supplementary material for: Simultaneous targeting of linked loci in mouse embryos using base editing
Source: Sci Rep. 2019 Feb 7;9:1662. doi: 10.1038/s41598-018-33533-5 (PMC6367434; doi:10.1038/s41598-018-33533-5)
Supplement: Supplementary file 1 — Dataset 1 [file 41598_2018_33533_MOESM1_ESM.pdf]

# Simultaneous targeting of linked loci in mouse embryos using base editing

Hye Kyung Lee[1,7], Michaela Willi[1], Harold E Smith[2], Shannon M. Miller[3-5], David R. Liu[3-5], Chengyu Liu[6] and Lothar Hennighausen[1,7]

## Supplementary Figure legends

**Supplementary Fig. 1** Simultaneous targeting of two linked genomic loci using CRISPR/Cas9 genome editing. (**a**) Summary of data obtained from founder mice in which 18 kb, 9 kb, and 22 kb loci between two sites were targeted by Cas9 and two sgRNAs, respectively. The sgRNA sequences are underlined and PAM sites are highlighted in brown. Deletions are shown as dash lines. The frequency column illustrates the percentage of large deletions obtained from mutant mice generated by simultaneous injection with two sgRNAs by CRISPR/Cas9 genome editing. (**b**) List of studies on CRISPR/Cas9 genome editing of the mouse germline and summary of large deletions caused by CRISPR/Cas9 and multiple sgRNA injection into mouse zygotes.

**Supplementary Fig. 2** The enhancer landscape of the *Csn2* and *Csn1s2a* locus using ChIP-seq analysis for transcription factor binding (STAT5, GR, ELF5 and MED1) and the presence of H3K27ac marks.

**Supplementary Fig. 3** Whole genome sequence analysis. (**a**) Number of predicted off-targets for two sgRNAs. (**b**) Number of total SNPs and indels as well as the number of mutations located at potential off-target sites identified in two base-edited mice, a female (F885) and a male (M888).

# Supplementary Fig. 1

## a

| Target | Large deletion | Frequency |
|---|---|---|
| *Wap-Ramp3* locus | 18kb | 11/14 (79%) |
| | WT ATATATGGTCAGCATATAAAG**CCA**GCAGACTTCCCAGATCTCCC......**CCT**GCCCTCAGTTAGCTTCAGAC | |
| | F591 ATATA---------------------------------------......---------AGTTAGCTTCAGAC | |
| | F592 ATATATGGTCAGCATATAAA------------------------......------CTCAGTTAGCTTCAGAC | |
| | F593 ATATATGGTCAGCATATAAA------------------------......---------AGTTAGCTTCAGAC | |
| | F595 ATATATGGTCAGCATATAAAGCCAGC------------------......----------------TTCAGAC | |
| | F597 ATATA---------------------------------------......------CTCAGTTAGCTTCAGAC | |
| | F598 ATATATGGTCAGCATATAAAGCCAG-------------------......----------------------- | |
| | M599 ATATATGGTCAGCATATAAAGCCA--------------------......-------------AGCTTCAGAC | |
| | M601 ATATATGGTCAGCATAT---------------------------......----------------------- | |
| | M1482 ATATATGGTCAGCATATAAAGC----------------------......-----------TTAGCTTCAGAC | |
| | M1483 ATATATG-------------------------------------......----------------------- | |
| | F1484 --------------------------------------------......-------------AGCTTCAGAC | |
| *Csn* super-enhancer locus | 9kb | 15/23 (65%) |
| | WT ACCT**CCT**GAGACCCTACATGCTTAGCG......**CCT**GTCTAGTGGAAGAACACCCATCTAA | |
| | F6385 ACCTCCTG-------------------......----------GGAAGAACACCCATCTAA | |
| | F6386 ---------------------------......---------------AACACCCATCTAA | |
| | M6390 ---------------------------......-------------------CCCATCTAA | |
| | M6392 ACCTCCTG-------------------......---------------AACACCCATCTAA | |
| | M6393 ACCTCCTGA------------------......--------------GAACACCCATCTAA | |
| | M6394 ACCTCTAACCTCCT-------------......---------------------------- | |
| | F6395 ACCTCCTGA------------------......------TAGTGGAAGAACACCCATCTAA | |
| | F6396 ACCTCCTG-------------------......---------------------------- | |
| | M6398 ACCTCCTGAG-----------------......------TAGTGGAAGAACACCCATCTAA | |
| | F6426 ---------------------------......---------------------------- | |
| | F6427 ---------------------------......---------------------------- | |
| | M6429 ACCTC----------------------......---------TGGAAGAACACCCATCTAA | |
| | M6430 ---------------------------......---------------------------- | |
| | M6432 ACCTCCTGAG-----------------......---------TGGAAGAACACCCATCTAA | |
| | M6433 ACCTCCTGAG-----------------......---------TGGAAGAACACCCATCTAA | |
| PMID 28561021 (D-1/2/3/4/5) | 22kb | 9/10 (90%) |
| | WT CTCTGAGTTTCCCATATGAACTC**AGG**AGACCCA......TATGTGT**CCT**TGGGTGTATGTAAGTAAGTGTGT | |
| | F2215 ---------------------------------......--------------------------------- | |
| | F2216 CTCTGAGTTTCCCATAT----------------......--------------TGTATGTAAGTAAGTGTGT | |
| | F2217 ---------------------------------......----------------TATGTAAGTAAGTGTGT | |
| | F2218 CTCTGAG--------------------------......--------------------------------- | |
| | M2219 ---------------------------------......TATGTGTCC------------------------ | |
| | M2220 ---------------------------------......-------------GTGTATGTAAGTAAGTGTGT | |
| | M2221 CTCTGAGTTTCCCATATG---------------......-------------------GTAAGTAAGTGTGT | |
| | M2222 ---------------------------------......--------------------------------- | |
| | M2223 ---------------------------------......--------------------------------- | |

## b

| PMID | Large deletion | Frequency |
|---|---|---|
| 26742453 | 9 kb | 3/30 (10%) |
| 23997119 | 10 kb | 9/27 (33%) |
| 25137067 | 23 kb | 4/25 (16%) |
| 25137067 | 23 kb | 3/9 (33.3%) |
| 25803037 | 65 kb | 3/14 (21%) |
| 26742453 | 65 kb | 13/81 (16%) |
| 26620761 | 95 kb | 2/20 (10%) |
| 27396308 | 0.5 M | 6/27 (22%) |

# Supplementary Fig. 2

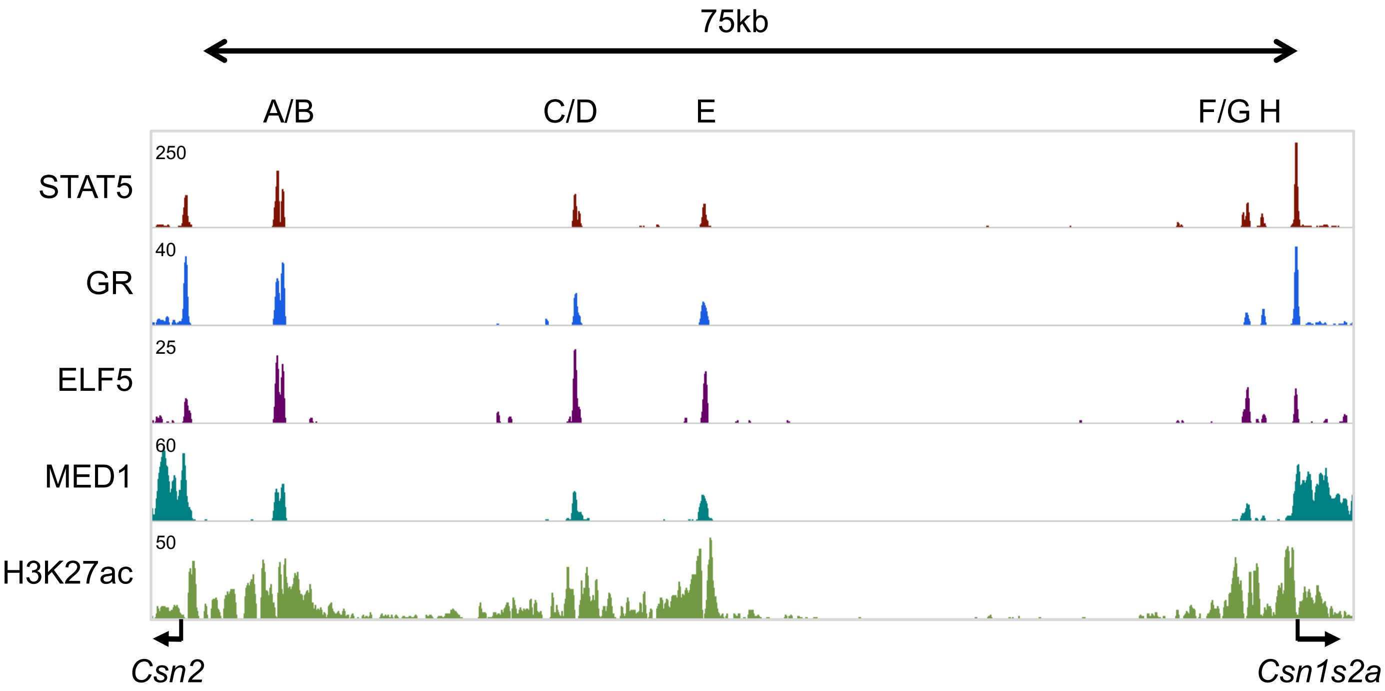

# Supplementary Fig. 3

## a

| Base editor | Protospacer | PAM | Number of Potential off-targets |
|---|---|---|---|
| VQR-BE3 | GAGTTCAAAGAAGGCAGGAA | AGAG | 434 |
| BE4 | CTTCCTTGTTCACACCCTTT | GGG | 143 |

## b

| | F885 | M888 |
|---|---|---|
| Number of total SNPs | 12,310 | 12,705 |
| Number of total INDELs | 182 | 172 |
| Number of SNPs at potential off-target sites | 0 | 0 |
| Number of INDELs at potential off-target sites | 0 | 0 |
